# Quantitative and qualitative condylar changes following stabilization splint therapy in patients with temporomandibular joint disorders with and without skeletal lateral mandibular asymmetry: a cone beam computed tomographic study

**DOI:** 10.1186/s12903-024-04119-7

**Published:** 2024-03-21

**Authors:** Mazen Musa, Riham Awad, Salma Izeldin, Yunshan Zhao, Hao Wu, Lu Wang, Saba Ahmed Al-hadad, Bdr Sultan Saif, Madiha Mohammed Saleh Ahmed, Xi Chen

**Affiliations:** 1 Department of Stomatology, The First Affiliated Hospital of Xi’an Jiaotong University, Xi’an, Shaanxi 710061 People’s Republic of China; 2 Department of Orthodontics, Al Tegana Dental Teaching Hospital, Faculty of Dentistry, University of Science and Technology Omdurman, Khartoum, Sudan; 3 Department of Pediatrics Dentistry, College of Stomatology, Xi’an Jiaotong University, Xi’an, Shaanxi People’s Republic of China; 4 Department of Pediatrics Dentistry, Faculty of Dentistry, International University of Africa, Khartoum, Sudan; 5 Department of Orthodontics, Faculty of Dentistry, Karary University, Omdurman, Khartoum Sudan; 6 Department of Orthodontics and Dentofacial Orthopedics, Faculty of Dentistry, Ibb University, Ibb, Republic of Yemen; 7 Department of Orthodontics, Faculty of Dentistry, Aden University, Aden, Republic of Yemen

**Keywords:** Chin deviation, Cone beam computed tomography (CBCT), Facial asymmetry, Bone mineral density, Craniomandibular disorders

## Abstract

**Background:**

Temporomandibular disorders (TMDs) encompass pain and dysfunction in the jaw, muscles, and adjacent structures. This study aimed to explore the quantitative (condylar position, morphology) and qualitative (bone mineral density (BMD)) therapeutic outcomes following a stabilization splint (S.S.) therapy in adult patients diagnosed with TMD (Arthralgia) with/without lateral mandibular asymmetry (MA) using cone beam computed tomography (CBCT).

**Methods:**

In this retrospective clinical study, 60 adult TMD patients who received S.S. therapy were enrolled and allocated into the TMD group (TMDG) and TMD with MA group (TMD + MAG). The diagnosis was made according to the Diagnostic Criteria for TMD (DC/TMD) AXIS I. MA was measured from the mid-sagittal plane to the Menton point. CBCT was used to scan the temporomandibular joints pre- (T0) and post- (T1)-treatment for three-dimensional analysis. Intra- and intergroup statistical comparisons were performed using the Wilcoxon signed ranks and the Kruskal‒Wallis test.

**Results:**

For quantitative comparisons, there was a statistically significant difference between T0 and T1 in the joint spaces of TMD + MAG (anterior, superior, posterior, and coronal lateral on the deviated side as well as in the superior, coronal medial joint space of the contralateral side). Morphologically, the deviated side had a narrower condylar width, reduced condylar height, and a steeper eminence angle. In contrast, the contralateral side tended to have a greater condylar length. For qualitative measurements, BMD also showed statistical significance between T0 and T1 in the majority of the condyle slopes (AS, SS, PS, and LS on the deviated side and in AS and MS on the contralateral side) of TMD + MAG. Additionally, only the AS and PS showed significance in TMDG.

**Conclusion:**

Multiple joint space widening (AJS and CMS) and narrowing (SJS, PJS, and CLS) could characterize the deviated side in TMD + MA. Factors like narrower condylar width, reduced condylar height, and steeper eminence angle on the deviated side can worsen TMD + MA. Proper alignment of the condyle-disc position is essential for optimal function and load distribution, potentially affecting bone mineral density (BMD). MA plays a prominent role in disturbing bone densities. S.S. therapy shows more evident outcomes in TMD + MAG (on the deviated side compared to the contralateral side) than the TMDG.

**Supplementary Information:**

The online version contains supplementary material available at 10.1186/s12903-024-04119-7.

## Background

Temporomandibular disorders (TMDs) encompass a wide range of conditions that impact the masticatory system and adjacent structures [1]. TMDs affect 5-12% of the population [2], with higher prevalence in women (30%) than men (21%) [3]. Cartilage integrity loss, pain, disc displacement, changes and loss of synergy of the condyle–disc–eminence complex, popping, clicking, limited opening, mandibular deviation on opening and closure, muscle discomfort, headaches, and earaches are symptoms of TMDs [4]. The etiology and pathophysiology of TMD are not well understood; however, it is widely accepted that it is a multifactorial phenomenon [5]. The reported prevalence of TMD is heavily influenced by various factors, including the choice of diagnostic criteria, clinical examination procedures, characteristics of the study population, and the expertise of the investigators [6]. The diagnosis of TMDs has evolved over time, with the introduction of the Diagnostic Criteria for Temporomandibular Disorders (DC/TMD) in 2014 [2], replacing the Research Diagnostic Criteria for Temporomandibular Disorders (RDC/TMD) [7].

Traditional two-dimensional (2D) radiography was the primary temporomandibular joint (TMJ) imaging method. However, due to the overlap of nearby structures and the limited sensitivity to changes in both condylar and temporal bone components, this 2D approach is unreliable [8]. The development of three-dimensional (3D) and magnetic resonance imaging (MRI) imaging made it possible to analyze the TMJ much more precisely [9]. Cone beam computed tomography (CBCT) exposes patients to less radiation than conventional computed tomography CT. Its high-resolution imaging can reach excellent performance in terms of accuracy when examining the TMJ [10].

Mandibular asymmetry (MA) is a common craniofacial deformity characterized by the lateral deviation of the mandible’s midline [11]. It can manifest in different parts of the face, with varying frequencies (upper, middle, and lower thirds of 5%, 36%, and 74%) [12]. MA can lead to symptoms such as pain, joint noises, and limited jaw movement [13]. The causes of MA can be attributed to various factors, including pathogenic, traumatic, functional, or developmental reasons [14]. These factors can be acquired postnatally or inherited prenatally [15]. Research suggests that early detection and intervention during mixed dentition can prevent the noticeable progression of mandibular deviation as the patient ages [16]. Typically, clinical and radiographic examinations are used to make the traditional diagnosis of MA. Frontal cephalography, submentovertex, and panoramic X-rays are the most often utilized images; however, since 3D allows us to view craniofacial bones from various angles, they provide more accurate visualization than traditional 2D radiographs [17]. Facial Asymmetry is prevalent in TMD patients with internal derangement (ID) [18]. TMD is linked to disrupted facial skeleton growth, such as MA, in rabbits and humans [19, 20]. Studies have suggested that MA could be an etiopathologic component in TMD [21, 22]; additionally, there is a belief that TMD and MA are related [23]. Disc displacement without reduction (DDwoR) induced through surgical intervention reduces the mandibular ramus length on the ipsilateral side [24]. Likewise, there is a potential association between ID and abnormal growth of the facial skeleton, including conditions such as retrognathia and MA [20].

TMD treatment approaches include conservative treatment with therapeutic exercises and education on habits and stress reduction. Occlusal splint therapy is used to restore jaw alignment. Massage therapy and manual therapy target myofascial pain and trigger points. Other physiotherapeutic techniques, such as biofeedback and ultrasound therapy, are employed. Pharmacotherapy includes myorelaxants, NSAIDs, analgesics, and psychotherapy (antidepressants). Surgical procedures like arthrocentesis may be used in severe cases. Acupuncture and alternative therapies or combinations can also be considered [25]. There remains a lack of agreement regarding the specific level of Asymmetry that should be deemed normal for patients preparing for surgery. One of the most conservative treatments for TMD from different origins (Myogenic and Arthrogenic) is using a stabilization appliance or stabilization splint (S.S.) [26]. Although the literature on its effectiveness in treating TMD is controversial, a recent meta-analysis suggested that S.S. could have a key role in treating TMDs [27]; another study found no evidence of the splint’s effectiveness in treating TMD [28]. In addition, there is no evidence to support or invalidate the use of S.S. for TMD treatment [29]. S.S. can improve the facial Asymmetry of patients with TMD and MA to a certain extent through mandibular rotation around the midsagittal plane, making the mandible position move more to the middle of the face [30].

Asymmetries ultimately result in imbalanced occlusion, problems of masticatory muscles, and TMJ problems [31]. Patients with TMD are usually found to have extensive disc displacement on the asymmetrical side of their faces [32]. Likewise, the degree of MA is related to the severity of disc displacement, and patients with Menton deviation could be more disc-displaceable [33]. Clinical signs and symptoms of TMD in patients with MA are more prevalent than those without MA (35.3–85.7%) [34]. However, the precise association between TMD and the presence or absence of MA remains inadequately substantiated. A knowledge gap was observed regarding TMD pre-and post-treatment with S.S. in the presence and absence of MA and its effect on condyle position, morphology, remodeling, and their correlations.

The objective of the present study was to evaluate quantitative (condylar position, morphology) and qualitative (bone mineral density (BMD)) therapeutic outcomes following S.S. therapy in adult patients diagnosed with TMD (intra-articular joint disorders arhrogenic TMD) in the occurrences and absences of skeletal MA, using CBCT. Determining this link may benefit TMD and MA patients regarding diagnostic and treatment aspects.

## Methods

### Study design

The First Affiliated Hospital of Xi’an Jiao Tong University, China, ethics committee approved this retrospective clinical study (No. XJTU1AF2022LSK-027).

The primary outcome of our study was to investigate the quantitative and qualitative therapeutic outcomes of S.S. in individuals with TMD + MA (deviated and contralateral sides). Additionally, the secondary outcome was to compare the outcomes between individuals with TMD + MA and those with TMD only (right and left side).

## Participants

The study included patients who consulted the Department of Stomatology, First Affiliated Hospital of Xi’an Jiao Tong University, China, between July 2017 and January 2023 and were diagnosed with TMD (intra-articular joint disorders/Arthralgia) with and without MA. Additionally, informed consent was obtained from all patients involved in the study. The sample size was calculated using G*Power (V. 3.1.9.4), with an alpha value of 0.05 and a power of 80%, based on a pilot study in which the changes in the AJS mean for the deviated side and contralateral side were 1.87 ± 0.81 and 2.99 ± 1.88, respectively. The resulting sample size was a minimum of 29 patients for each group. This number was increased later to 30.

### Inclusion criteria

Adult patients > 18 y old, detailed medical and oral history, a full AXIS I DC/MD clinical examination, meeting one of the following TMD diagnoses: Intra-articular Joint Disorders: Disc Displacement with Reduction (DDwR) or Disc Displacement with reduction, with intermittent locking (DDwRIL), Arthralgia, whose treatment plan included maxillary S.S. with/without visible skeletal mandibular asymmetry, with full permanent dentition, clear radiographic (CBCT images one pre and one post-S.S. treatment) images allowing diagnosis of MA and show both condyles.

### Exclusion criteria

Patients with a history of congenital or developmental disorders (unilateral condylar hypoplasia or hyperplasia); recent TMJ injury or surgery; rheumatoid arthritis and other autoimmune diseases affecting TMJ idiopathic condylar resorption; osteoarthritis (OA); systemic diseases that may affect the masticatory system; under medication affecting bone metabolisms, such as calcitonin and hormone or other systemic diseases; patients who had received treatment for TMD prior to the study, history of orthodontic and orthognathic treatment; prosthetic replacement of teeth (partial or complete denture); pregnancy; radiotherapy; and patients treated with other types of splints.

## Instruments

### For TMD diagnosis

#### The symptom questionnaire (DC/TMD SQ) AXIS I

The DC/TMD SQ tool was used to gather information about a patient’s symptoms [2]. In order to reach a diagnosis, patients were expected to report pain in the relevant anatomical regions. The pain experienced should exhibit variability in response to functional activities and enable the identification of familiar pain when pressure is applied to the affected area during palpation.

#### Clinical examination according to AXIS I DC/TMD

The clinical examination was performed according to the DC/TMD Examination Protocol [2]. Two well-trained operators conducted the clinical examination under the direct supervision of an experienced TMD specialist who evaluated all cases. Furthermore, before the research commenced, the three operators calibrated to the specialist’s measurements to ensure accuracy.

TMD diagnosis was made based on the DC/TMD Diagnostic Decision Tree available online (https://ubwp.buffalo.edu/rdc-tmdinternational/tmd-assessmentdiagnosis/dc-tmd-translations/) and, accordingly, the Diagnostic Criteria Table. DC/TMD SQ and the DC/TMD Examination Protocol were incorporated into the TMD diagnosis. The diagnoses of intra-articular joint disorders were made based on the clinical findings.

### For MA diagnosis

**Fig. 1:**
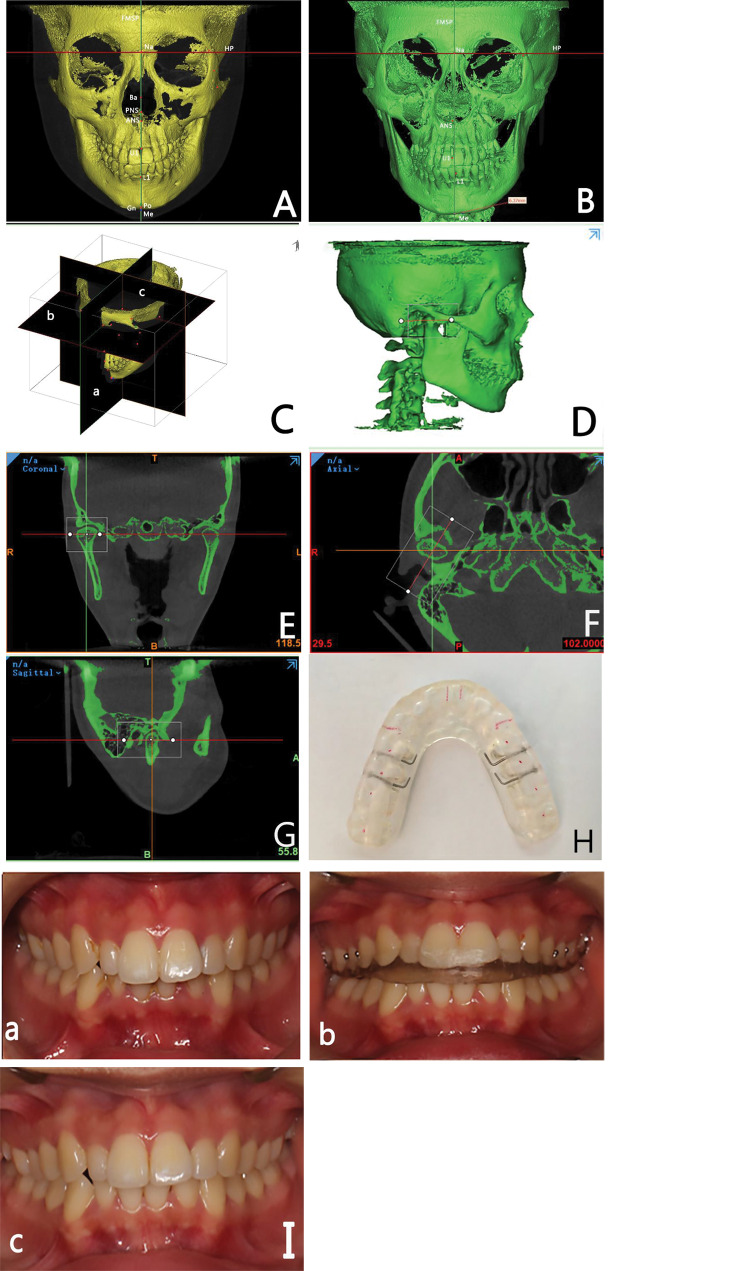
Anatomical landmark measurement, reference planes, and mandibular image processing showing (**A**) Frontal view of the TMD group; (**B**) Frontal view of the TMD + Mandibular Asymmetry group with amount of Menton deviation measured; **C**) Isometric view **a**: The midsagittal plane is formed by the nasion, sella, and basion point. **b**; The horizontal plane is formed by the right and left porions and the right orbitale. **c**; The sella point forms the vertical plane and is perpendicular to the horizontal and midsagittal planes. **2)** (**D**) A re-slice of the condyle in a three-dimensional view; (**E**) the coronal view; (**F**) the axial view; (**G**) the sagittal view. **3)** (**H**) Stabilization Splint (S.S.): Hard acrylic full coverage splint will be fitted to the upper arch and balanced to centric relation occlusion with anterior guidance on anterior teeth in red color; (**I**) **a**: before S.S. therapy; **b**: during S.S.; **c**: after S.S. A = anterior direction, P = posterior direction, T = top direction, B = bottom direction, R = right direction, L = left direction

Menton deviation was evaluated through a frontal three-dimensional assessment of the Asymmetry via CBCT radiograph; the mandibular deviation from the Menton point to the mid-sagittal plane (MSP) > 2 mm was used as the cutoff point between the 2 groups. Their MA was quantified by measuring the degree of menton deviation; anatomical landmark measurements and reference planes were based on our previous work [26, 30] (Table 1; Fig. 1 (A, B, C)).

**Table 1:** Definitions of the selected anatomical landmark and reference planes

Abbreviation	Measurement parameters	Definition
**Anatomical Landmark**		
(Me)	Menton	The most inferior midpoint on the symphysis
(Gn)	Gnathion	The midpoint of the symphysis
(Pg)	Pogonion	The most anterior and midpoint on the symphysis of the mandible
(L1)	L1	Midpoint of the lower incisor edge.
(U1)	U1	Midpoint of the upper central incisors edge
(ANS)	Anterior Nasal Spine	The maxillary anterior nasal spine’s most anterior point
(Na)	Nasion	Anterior and superior frontonasal suture
(Ba)	Basion	The foramen magnum’s inferior-anterior margin in the skull base midline
(S)	Sella	The center point of the pituitary fossa is in the middle cranial fossa in sagittal and axial views.
(Or)	Orbital	The midpoint of the infraorbital margin.
(Po)	Porion	the most outer and superior bony points of the external acoustic meatus.
**Reference Planes**		
F/MSP	Facial/midsagittal plane	The plane constructed by (N), (BA), and (ANS) passes through (N) as the coordinate origin.
HP	Horizontal plane	A plane parallel to Or-FMSP passes through (N) as the coordinate origin.
CP	Coronal plane	A plane parallel to Or-FMSP passes through (N) as the coordinate origin.

### For qualitative and quantitative radiological assessment of the TMJ

CBCT imaging was used and acquired (KaVo Company, Germany); the applied parameters were set at 120 kV, 5 mA, a field of view (23 cm × 17 cm), and 17.8-s exposure time, with a voxel size of 0.3 mm and a slice thickness of 2 mm; all images were obtained under the same conditions by the same experienced radiologist. Patients were asked to sit and place their heads in the center of the headrest and then positioned parallel to the floor with the Frankfurt plane. Afterward, the patients were told to bite their teeth into the maximum intercuspal position (MIP), and the center beam was lined up with the sagittal plane. The CBCT scan data were transferred into Digital Imaging and Communication in Medicine (DICOM) file format and then imported into Mimics 21.0 software (Materialize Company, Belgium) for 3D reconstruction. After measuring MA, the mandibles were not separated from the whole image. The CBCT evaluations were conducted at 2-time points, pre-treatment T0 and post-treatment T1, to observe included groups of bony alterations in the condylar surface.

The deviated, contralateral, left, and right sides of the TMJs were evaluated independently for each patient. The TMJ was reoriented to reference planes, and images were resliced to identify the axial view, make the sagittal line perpendicular to the long condyle axis, and pass through the condyle center (Fig. 1D, E, F, G).

Sixty adult TMD patients met all the above inclusion criteria and were then classified into two groups with and without MA. Group one: 30 TMD patients (TMDG) relatively symmetrical patients whose MA was defined as a Menton deviation less than 2 mm (MSP nearly coincided with the chin midpoint (Menton)). Group two: 30 TMD patients who presented both TMD and observed MA (TMD + MAG).

### Treatment protocol

Based on previous work performed by our team, the study involved a multi-appointment treatment protocol for patients from both groups; please refer to [26] for a more detailed explanation. Clinical and radiographic examinations CBCT evaluation (at T0 to get the baseline measurements) were conducted during the first appointment to assess TMD symptoms and mandibular range of motion. The patients were evaluated for pain, noise, and limitations in mandibular movement.

At the second appointment, the patients were informed about their diagnosis based on the DC/TMD criteria. Initial records included upper and lower alginate impressions, a maximum intercuspation (MI) wax bite, and a preliminary 2-piece Roth power-centric relation (CR) bite registration using Delar blue wax following neuromuscular deprogramming, performed using a manual bilateral manipulation technique. A face bow was used to establish the relationship between the upper and the lower jaw, which was then transferred to a semi-adjustable articulator (AD 2®). A Measures Condyle Displacement device (MCD) evaluated the horizontal and vertical condylar positions (CP). The condyle displacement index shows that the MI-CR condyle displacement exceeds the physiological range MCD value of vertical dimension > 1 mm and transverse dimension > 0.5 mm. CP measurements were made on all casts, pre-treatment and post-treatment, to record the positional changes of the condylar axes from MI to CR.

During the third appointment, the maxillary S.S. (full coverage CR appliance) fabricated in a colorless thermopolymerized hard acrylic resin of 3 mm thickness was installed and adjusted for occlusal contacts (Fig. 1 (H, I)). The patients were instructed to wear the splints for at least 20 h daily, except while eating and brushing their teeth. Regular check-ups were conducted to monitor symptoms, joint area palpation, muscle tenderness, and splint readjustments if necessary [26]. During the fourth appointment, occlusal contacts were reassessed, and follow-up intervals were scheduled at 15, 30, and 60 days. The S.S. was gradually reduced until MIC was achieved [26].

The treatment duration ranged from 6 to 12 months, with an average of 9.1 months. No medication or physical therapy was administered, and treatment evaluation included patient reports, re-assessment, and improvement in TMD symptoms during follow-up visits. Eventually, the patients underwent a second DC/TMD clinical test, SQ, followed by another CBCT evaluation at the last appointment (at T1 to measure the intervention changes) after discontinuing using S.S.

### Quantitative outcomes assessment

**Fig. 2:**
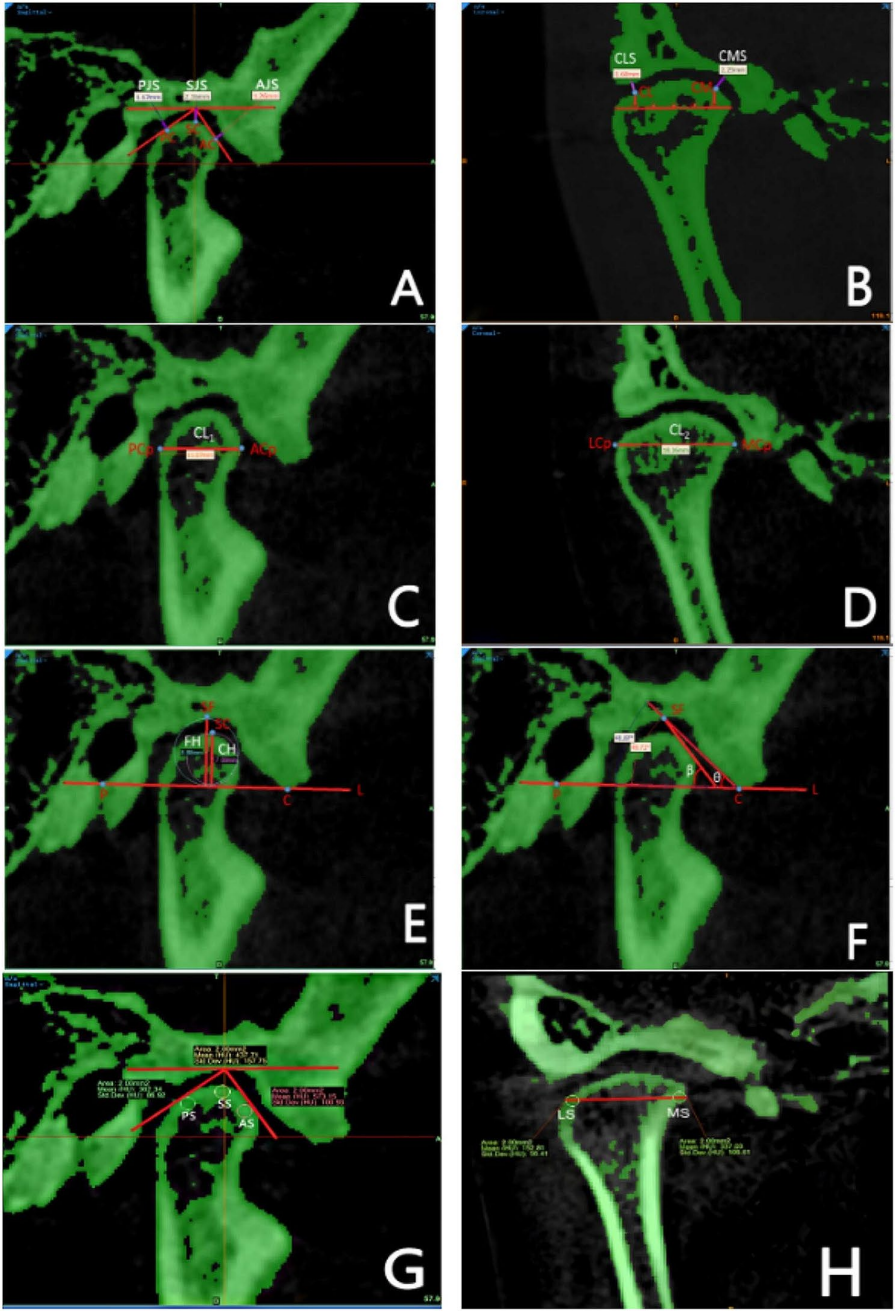
Quantitative and Qualitative Measurements of TMJ: Assessing Joint Spaces, Morphology, and Bone Mineral Density. **1)** Quantitative measurements: Assessing Joint Spaces (**A**) AJS: anterior joint space, SJS: superior joint space, PJS: posterior joint space; (**B**) CMS: coronal medial space, CLS: coronal lateral space. TMJ morphology measurement: (**C**) CL1: condyle anteroposterior diameter; (**D**) CL2: condyle mediolateral diameter, (**E**) CH: condyle height; FH: Fossa height; (**F**) β: condylar slope, θ: the inclination of the articular eminence. **2)** Qualitative measurements (**G**) The bone mineral density of the condyle in the three selected sites in the sagittal plane AS: anterior slope, SS: superior slope, PS: posterior slope; (**H**) the bone mineral density of the condyle in the coronal plane MS: medial slope, LS: lateral slope. A = anterior direction, P = posterior direction, T = top direction, B = bottom direction, R = right direction, L = left direction

The linear measurements of radiographic joint spaces in the sagittal plane were measured in millimeters, according to the Kamelchuk method (anterior joint space “AJS,” superior joint space “SJS,” and posterior joint space “PJS”) [35]. Meanwhile, the coronal plane followed the Ikeda method (coronal medial space “CMS” and coronal lateral space “CLS”) [36] (Table 2; Fig. 2 (A, B)).

**Table 2:** Definitions of the selected TMJ measurements

Abbreviation	Measurement parameters		Definition
**Quantitative measurements**			
AJS	Anterior joint space	(mm)	The vertical distance from the anterior-most mandibular condyle point (ACp) to the glenoid fossa.
SJS	Superior joint space	(mm)	The vertical distance from the most superior condyle point (SCp) to the most superior point of the glenoid fossa.
PJS	Posterior joint space	(mm)	The vertical distance from the posterior-most mandibular condyle point (PCp) to the glenoid fossa.
CMS	Coronal medial joint space	(mm)	The vertical distance from the condyle’s coronal medial point (CMp) to the glenoid fossa.
CLS	Coronal lateral joint space	(mm)	The vertical distance from the condyle’s lateral coronal point (CLp) to the glenoid fossa.
CL _1_	Condyle length	(mm)	The horizontal distance from the posterior-most condylar point (PCp) to the anterior-most condylar point (ACp).
CL _2_	Condyle width	(mm)	The horizontal distance from the medial condyle point (MCp) to the lateral condyle point (LCp).
CH	Condyle height	(mm)	The vertical distance from the most superior aspects of the condyle (SCp) to the reference line (L).
FH	Fossa height	(mm)	The vertical distance from the highest point of the fossa (SF) to the reference line (L).
β	The slope of the anterior condyle	( ° )	The angle formed between the line passing the tangent of the anterior slope of the condyle to point (SF) and the reference line (L).
θ	The inclination of the articular eminence	( ° )	The angle formed between the line passing through the tangent of the anterior wall of the articular eminence to point (SF) and the reference line (L).
β-θ	Condylar process - articular eminence relationship	( ° )	The difference between the slope of the anterior condyle and the tangent of the anterior wall of the articular eminence.
**Qualitative measurements**			
AS	Anterior slope	(Hu)	Anterior cortical bone density was measured in an area of 2mm^2^ ellipse in shape bone tissue representing the anterior slope, which was determined on the anterior-most mandibular condyle point (ACp).
SS	Superior slope	(Hu)	Superior cortical bone density was measured in an area of 2mm^2^ ellipse in shape bone tissue representing the superior slope which was determined on the superior condyle point (SCp).
PS	Posterior slope	(Hu)	Posterior cortical bone density was measured in an area of 2mm^2^ ellipse in shape bone tissue representing the posterior slope which was determined on the posterior-most mandibular condyle point (PCp).
MS	Medial slope	(Hu)	Medial cortical bone density was measured in an area of 2mm^2^ ellipse in shape bone tissue representing the Medial slope, which was determined on the condyle’s coronal medial point (CMp).
LS	Lateral slope	(Hu)	Lateral cortical bone density was measured in an area of 2mm^2^ ellipse in shape bone tissue representing the Lateral slope, which was determined on the condyle’s coronal medial point (CLp).

The determining quantitative landmarks and reference line for condyle morphology as described by Hilgers [37] (condyle length “CL 1,” width “CL 2,” height “CH,” fossa height “FH,” slope of the anterior condyle “β,” inclination of the articular eminence “θ,” and condylar process - articular socket relationship (β-θ)) are described in Table 1 and presented in Table 2; Fig. 2 (C, D, E, F).

### Qualitative outcomes assessment

The qualitative landmarks were determined according to the Kamelchuk method [35] (All data sets were subjected to Hounsfield unit calibration within the micro-CT program and standardized to achieve a consistent threshold difference to restore the contrast limits. A bone threshold value range of 226 to 3071 HU was chosen. Using Mimics software function Density in Ellipse, a round bone tissue with an area of 2 mm^2^ was selected in the sagittal plane (the anterior slope “AS,” superior slope “SS,” posterior slope “PS”). Meanwhile, in the coronal plane (medial slope “MS” and lateral slope “LS”), the condyle center is adjacent to the correct sagittal position. The bone density of ten continuous sections (thickness of 0.3 mm) was measured, and the average value was finally taken to represent the unit bone density of each slope of the condyle [26]. Table 2; Fig. 2 (G, H) describe mandibular skeletal measurements for qualitative outcomes. This study adopts relative values for BMD for the qualitative measures, making the measurement method highly reproducible [26]. The MIMICS software bone threshold was used to identify bone tissue automatically; it can accurately locate the condyle cortical boundary through the three-dimensional structure to avoid the interference of measurement errors. The quantitative and qualitative TMJ landmark definition description is provided in [Additional file 1].

### Statistical analysis

Statistical analysis was performed using SPSS 25.0 software (IBM, Chicago Inc., US). The measurements of CBCT were re-estimated by two different observers who re-analyzed the cases within two weeks to ensure intra- and inter-examiner reliability of the measures in 20 randomly selected patients. After data assessment for normality showed that the data did not obey a normal distribution using Shapiro‒Wilk’s test, the Wilcoxon signed ranks test was performed to test the statistical significance of the mean changes between pre-and post-treatment measurements in the same group. For intergroup comparisons, the Kruskal‒Wallis test was used. The mean and standard deviations were calculated using 0.05 as the significance level.

Furthermore, an analysis of effect size measures was performed using Cohen’s d test.

## Results

A total of 60 TMD patients treated with S.S. participated in this study, aged between 18 and 38 years, with a mean age of 28 and 2 months in the TMDG and 28 and 5 months in the TMD + MAG with a total of 66.67% women and 33.33% men (higher prevalence of women than men patients with MA and TMD). (No significant differences were observed regarding age (*p-*value = 0.136) and sex (*p-*value = 0.107).

For the TMDG, the MA mean measurement was 1.2 mm ± 0.5 (with a minimum of 0.5 mm and a maximum of 2 mm), while for the TMD + MAG, the mean measurement of MA was 5.13 mm ± 2.53 (with a minimum of 3.50 mm and maximum of 12.5 mm). A notable statistical significance was observed regarding the Menton deviation (*p-*value = < 0.001) between the TMDG and TMD + MAG. The intra- and inter-observer reliabilities for all the TMJ landmark outcomes ranged from 0.88 to 0.95; more about reliabilities are provided in [Additional file 2].

Regarding the analysis of effect size measures (Cohen’s d) output, the significant values ranged from 0.67 to 0.21, suggesting that the effect size measures of significant p-value went from a medium to a small effect.

### Quantitative outcomes

#### Joint space

In the TMD + MAG, statistically significant differences were observed between pre-T0 and T1 post-treatment, specifically in the AJS, SJS, PJS, and CLS (*p-*value = 0.001; 0.025; 0.001; 0.037, respectively) on the deviated side, as well as the SJS CMS (*p-*value = 0.026; 0.031, respectively) on the contralateral side. Furthermore, intra-group differences were observed in AJS, SJS, PJS, and CMS (*p-*value = 0.001; < 0.001; 0.01; 0.02, respectively) between the deviated and contralateral sides at T0. For the TMDG, a statistically significant difference was observed in the right and left sides regarding the AJS between T0 and T1, in addition to the AJS in the inter-group comparison (*p-*value = 0.042; 0.034; 0.021, respectively) (Tables 3 and 4).

**Table 3:** Comparison of quantitative and qualitative measurements pre-and post-treatment in the TMD + MA group

Measurement standard		Deviated side (No = 30)		Δ (T1-T0)	p-value	Contralateral side (No = 30)		Δ (T1-T0)	p-value	Intra-group comparison	
		T0	T1			T0	T1			D-T0 vs. C-T0	D-T1 vs. C-T1
		Mean ± SD	Mean ± SD			Mean ± SD	Mean ± SD			***p*** **-value**	
**Quantitative measurements**											
AJS	(mm)	2.99 ± 0.83	2.55 ± 0.20	-0.44	0.001**	1.71 ± 0.73	2.20 ± 0.89	0.49	0.0521	*	-
SJS	(mm)	1.78 ± 0.46	1.98 ± 0.32	0.20	0.025*	2.38 ± 0.81	1.90 ± 0.60	-0.48	0.026*	*	-
PJS	(mm)	1.60 ± 0.84	2.29 ± 0.65	0.69	0.001**	2.76 ± 0.78	2.20 ± 0.70	-0.56	0.079	*	-
CMS	(mm)	2.35 ± 0.43	2.20 ± 0.78	-0.15	0.055	1.68 ± 0.35	2.07 ± 0.59	0.39	0.031*	*	-
CLS	(mm)	1.82 ± 0.54	2.59 ± 0.40	0.77	0.037*	2.50 ± 0.51	2.30 ± 0.58	-0.20	0.091	-	-
CL 1	(mm)	7.85 ± 0.82	8.43 ± 1.02	0.58	0.064	9.89 ± 1.15	10.57 ± 1.26	0.68	0.132	-	-
CL 2	(mm)	16.01 ± 2.54	20.26 ± 2.00	4.25	0.045*	19.20 ± 2.46	23.14 ± 2.34	3.94	0.026	*	-
CH	(mm)	8.60 ± 0.92	8.40 ± 0.98	-0.20	0.030*	7.32 ± 0.98	8.44 ± 0.94	1.12	0.308	*	-
FH	(mm)	7.90 ± 1.19	8.04 ± 1.44	0.14	0.607	7.70 ± 1.28	7.87 ± 1.31	0.17	0.076	-	-
β	(°)	55.33 ± 7.69	59.01 ± 6.55	3.68	0.318	63.87 ± 7.89	62.60 ± 7.90	-1.27	0.113	-	-
θ	(°)	46.25 ± 6.99	47.35 ± 5.84	1.10	0.441	47.35 ± 7.10	46.18 ± 6.47	-1.17	0.216	*	-
β - θ	(°)	9.08 ± 6.25	11.66 ± 6.57	2.58	0.614	16.65 ± 6.23	16.34 ± 6.90	-0.31	0.657	-	-
**Quantitative measurements**											
AS	(Hu)	300.82 ± 96.7	326.61 ± 103.8	25.79	0.016*	318.24 ± 124	349.61 ± 103.8	31.37	0.013*	*	*
SS	(Hu)	292.74 ± 79.5	311.45 ± 99.7	18.71	0.034*	290.95 ± 107.5	309.09 ± 125.4	18.14	0.042*	*	-
PS	(Hu)	330.65 ± 90.4	359.98 ± 107.2	29.33	0.002**	299.53 ± 87.9	318.59 ± 98.19	19.06	0.003**	*	-
MS	(Hu)	297.45 ± 83.1	314.78 ± 79.7	17.33	0.074	300.20 ± 91.4	320.33 ± 85.6	20.13	0.003**	*	-
LS	(Hu)	280.21 ± 78.3	300.01 ± 56 0.3	19.80	0.010*	289.87 ± 77.8	305.33 ± 99.7	15.46	0.069	*	-

MA: mandibular asymmetry; No: number of study sample per (joint); Δ: mean different; SD: standard deviation; mm: millimeters; °: degree; Hu: Hounsfield unit; T0: before treatment; T1: after treatment

*: *p*-value of < 0.05 statistically significant; **: *p* <0.01; ***: *p* <0.001; - not significant

#### Morphology

Regarding condyle morphology, in TMD + MAG, A statistically significant difference between pre-and post-treatment and intragroup comparisons at T0 was observed on the deviated side in CL2 and CH; additionally, the TMD + MAG shows a steeper eminence angle θ for the deviated side than for the contralateral side compared to TMDG, which was statistically significant (*p-*value = 0.045; 0.030; 0.011, respectively). No significant difference was observed in the TMDG’s bilateral eminence steepness in the TMD between the right and left sides group (Tables 3 and 4).

**Table 4:** Comparison of quantitative and qualitative measurements pre-and post-treatment in the TMD group

Measurement standard		Right side (No = 30)		Δ (T1-T0)	p-value	Left side (No = 30)		Δ (T1-T0)	p-value	Intra-group comparison		
		T0	T1			T0	T1			R-T0 vs. L-T0		R-T1 vs. L-T1
		Mean ± SD	Mean ± SD			Mean ± SD	Mean ± SD			***p*** **-value**		
**Quantitative measurements**												
AJS	(mm)	2.33 ± 0.65	2.06 ± 0.66	-0.27	0.042*	1.88 ± 0.52	1.66 ± 0.60	-0.22	0.034*	*	-	
SJS	(mm)	1.77 ± 0.75	1.96 ± 0.48	0.19	0.111	2.30 ± 0.44	2.60 ± 0.75	0.30	0.150	-	-	
PJS	(mm)	1.80 ± 0.92	2.02 ± 0.60	0.22	0.432	1.72 ± 0.56	1.90 ± 0.60	0.18	0.145	-	-	
CMS	(mm)	2.30 ± 0.62	2.08 ± 0.87	-0.22	0.064	2.43 ± 0.57	2.22 ± 0.89	-0.21	0.050	-	-	
CLS	(mm)	1.89 ± 0.70	2.10 ± 0.89	0.21	0.367	1.91 ± 0.64	2.09 ± 0.89	0.18	0.231	-	-	
CL 1	(mm)	9.54 ± 1.45	10.26 ± 1.64	0.72	0.051	9.70 ± 1.26	10.71 ± 1.15	1.01	0.005**	-	-	
CL 2	(mm)	17.30 ± 1.22	19.14 ± 1.12	1.84	0.086	18.00 ± 2.34	20.90 ± 2.46	2.90	0.077	-	-	
CH	(mm)	8.44 ± 0.92	8.50 ± 0.98	0.06	0.083	7.36 ± 0.80	8.44 ± 0.98	1.08	0.308	-	-	
FH	(mm)	7.69 ± 1.28	7.45 ± 1.44	-0.24	0.101	7.66 ± 1.44	6.88 ± 1.28	-0.78	0.521	-	-	
β	(°)	63.96 ± 7.91	62.60 ± 7.94	-1.36	0.920	63.80 ± 7.91	62.60 ± 7.94	-1.20	0.202	-	-	
θ	(°)	47.35 ± 6.84	46.24 ± 7.15	-1.11	0.362	47.06 ± 6.84	46.24 ± 7.15	-0.82	0.130	-	-	
β - θ	(°)	16.61 ± 6.57	16.36 ± 6.18	-0.25	0.093	16.74 ± 6.57	16.36 ± 6.18	-0.38	0.066	-	-	
**Quantitative measurements**												
AS	(Hu)	350.70 ± 97.2	379.73 ± 90.7	29.03	0.021*	329.33 ± 99.8	345.34 ± 100	16.01	0.045*	*	-	
SS	(Hu)	320.43 ± 89.6	349.59 ± 89.3	29.16	0.061	307.86 ± 80.7	330.95 ± 91.1	23.09	0.082	-	-	
PS	(Hu)	309.97 ± 70.8	329.06 ± 105.6	19.09	0.074	302.83 ± 76.2	320.09 ± 82.8	17.26	0.031*	-	-	
MS	(Hu)	310.57 ± 83.1	328.45 ± 79.7	17.88	0.053	300.72 ± 84.7	317.33 ± 81.7	16.61	0.091	-	-	
LS	(Hu)	270.88 ± 68.3	285.87 ± 56.3	14.99	0.102	262.46 ± 99.7	274.03 ± 99.4	11.57	0.076	-	-	

No: number of study sample per (joint); Δ: mean different; SD: standard deviation; mm: millimeters; °: degree; Hu: Hounsfield unit; T0: before treatment; T1: after treatment

*: *p*-value of < 0.05 statistically significant; **: *p* <0.01; ***: *p* <0.001; - not significant

### Qualitative outcomes

#### BMD

In terms of BMD, statistical significance was observed in the TMD + MAG regarding AS, SS, PS, and LS on the deviated side and in AS, SS, PS, and MS of the contralateral side pre- and post-treatment (*p-*value = 0.016; 0.034; 0.002; 0.010; 0.013; 0.042; 0.003; 0.003, respectively); moreover, all slopes for intra-group comparison were also significant at T0, while only AS remained significant for T1. Additionally, for the TMDG, the AS showed statistical significance on both the right and left sides in pre-post-treatment comparisons, as well as in intra-group comparisons at T0 (*p-*value = 0.021; 0.045; 0.04); furthermore, PS on the left side also showed statistical significance (*p-*value = 0.031) (Tables 3 and 4).

## Discussion

The present study aimed to explore the therapeutic outcomes of S.S. in adult patients with TMD (intra-articular joint disorders/Arthralgia) with/without MA using CBCT to assess quantitative (condylar position, morphology) and qualitative (BMD) measures.

Regarding the quantitative outcome, in TMD + MAG, the (SJS, PJS, and CLS) were narrower on the deviated side. This aligns with the study by Akahane et al. [38], which found narrow SJS. Endo et al. [39] found narrow joint space in the posterolateral section. In contrast, in this study, the contralateral side had wider PJS, SJS, and CLS; however, it was significant in SJS and CMS only, suggesting a downward and medial condyle position at T0. Meanwhile, Kawakami et al. [40] found the AJS to be narrower on the deviated side.

The findings for post-treatment T1, on the deviated side, are as follows: the AJS and CMS joint space averages were reduced while, simultaneously, SJS, PJS, and CLS were increased compared to pre-treatment T0. These changes suggest that the condyle on the deviated side was positioned upward, backward, and lateral at T0, probably due to the disc’s prolonged anterior and medial positioning, which may have been displaced. As a result of a displaced disc, the condyle will vertically adjust itself to fill that space occupied by the disc; furthermore, it moved downward forward and medially post-treatment T1, leading to the upward and lateral position of the contralateral side (contributing to MA improvement). The findings of T0 are in agreement with the findings of Alhammadi et al. [41], who reported that the condyle was in superior, posterior, and lateral positions, while Akahane et al. [38] suggested upward positioning. The findings of T1 are in agreement with [26, 30, 42].

In TMDG, a significant difference was observed between the right and left sides in the AJS pre- and post-treatment and in intra-group comparison, possibly due to asymmetrical disc position. However, no significance was observed in the intra-group comparison at T1, indicating that S.S. effectively balanced the joint space average.

Regarding the morphology, in TMD + MAG, the deviated side had a smaller condylar diameter (CL 1 and CL 2), which was significant in CL 2. Asymmetrical loading altered growth environment may explain this. The contralateral side was the largest, possibly due to excessive growth and muscle tension. Similar findings were reported in previous studies on patients with MA [34, 38, 43, 44].

Okur et al. [45] found a significant difference in condylar width between patients and controls. Seo et al. [46] found a narrower condyle width in ADDWR patients. Alhammadi et al. [41] found no significant difference in condyle width and length between TMD and non-TMD patients.

The deviated side having a smaller condyle increases the likelihood of disc displacement compared to the contralateral side. As internal derangement (ID) progresses, the condyle decreases in the mediolateral dimension, potentially leading to lateral pole resorption [46]. Collectively, these findings support a potential link between MA and disc displacement and changes in TMJ condyle size (CL 2), supported by the Kurita et al. study [47].

Significant side differences were observed in the morphology as reduced condylar height (CH) on the deviated side, while the contralateral side exhibited greater condylar length. Zhao et al. [48] suggested that condylar size reduction is an adaptive change to MA, influenced by muscle activity [49]. MA induces morphological and cellular changes in the condyle, synovial membrane, and masticatory muscle. Mechanisms such as VEGF protein overexpression and oxidative stress/nitric oxide imbalance may contribute to unbalanced TMJ loading [50]. However, there were no statistically significant differences in FH, contrasting with findings by Alhammadi et al. in TMD and non-TMD patients [41].

The TMD + MAG shows a steeper eminence angle (θ) for the deviated side than for the contralateral side, which was statistically significant. This potentially arises as an adaptation to the asymmetrical loading experienced by the TMJ, indicating that not only does the condyle undergo a remodeling process but also articular eminence to keep the anterior condylar process—articular eminence relationship in rhythm. This observation aligns with similar findings reported by [40]. While in the TMDG, it was insignificant.

Regarding qualitative outcomes, this study observed higher BMD for TMDG than TMD + MAG, suggesting that MA plays a prominent role in disturbing BMD, which will be expressed as a change in morphology. In TMD + MAG, the majority of contralateral side slopes had higher BMD (particularly AS, followed by MS) than the deviated side, except for PS. At the same time, the PS had the highest BMD, followed by AS, and LS had the lowest BMD pre-and post-treatment on the deviated side of the same group, which is consistent with the suggested abovementioned condyle movement. This assumes that the lower bone density before S.S. treatment was an explanation for having Arthrogenic TMD, and later, S.S. therapy improved bone density.

This study revealed that the SS-BMD was higher on the deviated side than on the contralateral side, which supports the theory that the deviated side was in an upward position. The posterior deflection of the condyle on the deviated side may explain the higher BMD of PS compared to AS. On the contralateral side, the AS-BMD was higher than the PS. This may be explained by the fact that the AS remains the main loaded surface during jaw movement; in contrast, the posterior deflection of the deviated condyle may let PS and SS be loaded. The lower bone density of the PS on the contralateral side shows that the PS endures lower strength than the deviated side. Another observation of this study regarding the treatment effect **(Δ)** is that PS had the highest treatment effect (T1-T0), followed by AS, and LS had the lowest treatment effect in the TMD + MAG. In the TMDG, AS had the highest treatment effect, followed by MS and LS, which were also the lowest.

Several studies have examined BMD in relation to TMD, and S.S. Musa et al. [26] found that S.S. improved condyle bone density more noticeably in the Arthralgia group than in the Myalgia group. Kim et al. [51] demonstrated bone surface remodeling in TMJ-OA patients with bone resorption and formation areas after S.S. therapy. Ok et al. [52] observed bone formation and cortical thickening in TMJ-OA patients undergoing S.S. treatment. Evaluating BMD in patients with mandibular asymmetry (MA), Lin et al. [53] found higher BMD on the deviated side, while Wen et al. [43] found higher BMD on specific points of the contralateral side. These findings support a relationship between asymmetrical jaw function and BMD.

**Fig. 3:**
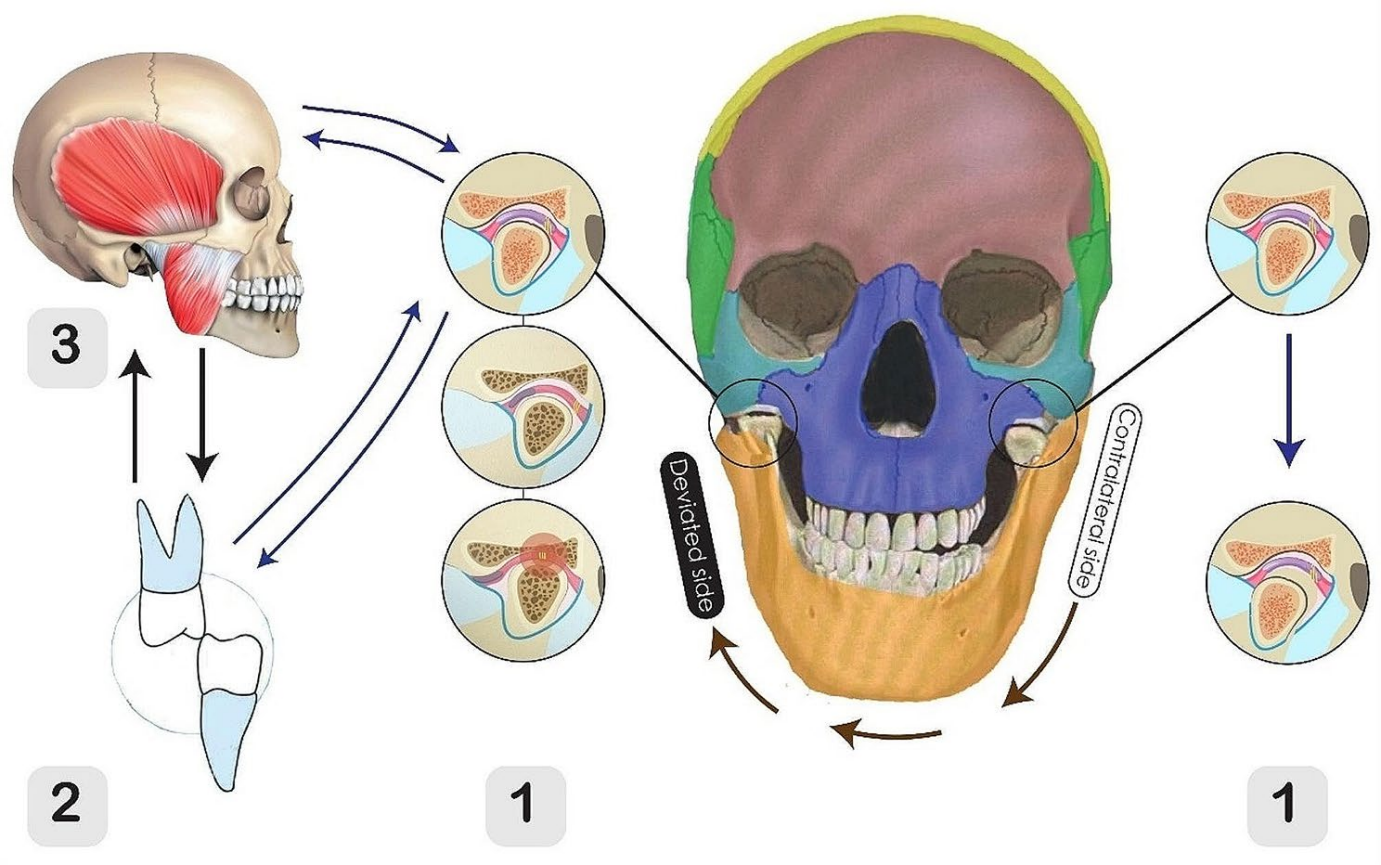
The possible effect of mandibular asymmetry (MA) on the stomatognathic system based on the degree of MA. **(1)** The disc and condylar changes on the deviated (backward, upwards, and laterally) and contralateral (downward forward and medially) sides. **(2)** Occlusal changes on the deviated (loss of maximum intercuspation or crossbite) and contralateral (increase in overbite) sides. **(3)** Regarding muscular changes on the deviated side, the maximum contraction of the muscle of mastication may be challenging to achieve; however, it may remain possible on the contralateral side, worsening skeletal mandibular asymmetry with soft tissue asymmetry

Dong et al. [54] state that persistent asymmetrical muscle activity is associated with TMJ and cervical pain. In cases of MA, an occlusal interference on the deviated side may develop, making the maximum contraction of the muscle of mastication challenging to achieve, which over time results in uni-lateral muscle use (muscle atrophy) on the deviated side compared to muscle overuse (hypertrophy) on the contralateral side. To address this, the authors suggest combining S.S. with myo-functional therapy to strengthen the weak muscles on the deviated side and reduce muscle imbalances. The gentle isometric jaw exercises can increase the strength of atrophied muscles [55], thereby influencing the growth environment of the condylar cartilage and gradually changing condylar morphology [40] (Fig. 3).

Based on our study, the intensity of disc displacement was quantitatively and qualitatively related to the amount of MA. Additionally, skeletal MA may be considered a potential risk factor warranting further investigation in the context of TMD etiology.

The results of the present study demonstrate a statistically significant difference in quantitative and qualitative S.S. therapeutic outcomes between TMD + MAG and TMDG and between the deviated and contralateral sides of the TMD + MAG, suggesting sufficient evidence to reject the null hypothesis.

This research holds significant importance in clinical practice; it contributes to the existing body of knowledge on TMD, MA, and splint therapy, as it provides a comprehensive assessment and elucidates the therapeutic outcome of S.S. on symptomatic populations affected by lateral MA and TMD from both qualitative and quantitative standpoints. S.S. for patients with TMD + MA is recommended as S.S.-induced quantitative and qualitative (positional, morphological, and BMD) therapeutic outcomes may contribute to MA improvement. Multiple joint space widening (AJS and CMS) and narrowing (SJS, PJS, and CLS), along with changes in size (CH and CL2) and a steep articular eminence, were identified as prominent features in TMD + MAG. These findings have diagnostic and prognostic implications for TMD. The study also suggests a potential association between mandibular asymmetry (MA), TMJ disc displacement, and condyle width (CL2). Clinicians should be mindful that MA can contribute to bone metabolism imbalances, affecting formation and resorption. When evaluating patients with abnormal condylar bone density, considering the possibility of underlying MA is crucial, making MA an indicator for potential exacerbation of TMD and serving as a prognostic factor. Furthermore, the initiation of early treatment may stop the disease from progressing.

No study is without limitations; some of the limitations in our study include a relatively small sample size. Furthermore, the assessment of disc position did not involve MRI, potentially influencing the accuracy of condylar movement evaluation within the TMJ, and long-term follow-up is missing. Future prospective studies should be conducted to address these limitations, employing both CBCT and MRI techniques to comprehensively evaluate TMJ bony structures and the articular disc while incorporating a pain-free control group. Additionally, assessing the TMJ at a later point T2, such as 6–12 months, is recommended to investigate whether observed changes revert to normal as patients return to their habitual MIP.

## Conclusions

- The joint space is important for diagnosing and prognosis of TMD; multiple joint space widening (AJS and CMS) and narrowing (SJS, PJS, and CLS) could characterize the deviated side of the condyle in the TMD and mandibular asymmetry (TMD + MA).
- A narrower condylar width (CL 2), reduced condylar height (CH), and a steeper eminence angle (θ) on the deviated side can potentially contribute to further exacerbations of the TMD sign and symptoms in patients with TMD + MA.
- Establishing and maintaining a properly aligned condyle-disc position in relation to the glenoid fossa is vital in ensuring optimal function and equitable distribution of loads, potentially influencing bone mineral density (BMD); this study suggests that mandibular asymmetry (MA) plays a prominent role in disturbing bone densities.
- The stabilization splints (S.S.) quantitative (position, morphology) and qualitative (bone mineral density (BMD)) therapeutic outcomes were more evident (on the deviated side than the contralateral) in the TMD + MA group. The significance of these outcomes was further highlighted in the TMD + MA group than in the TMD group. However, additional research is necessary to evaluate the long-term stability of S.S. treatment.

### Electronic supplementary material

Below is the link to the electronic supplementary material.

**Supplementary Material 1:** Shows the quantitative and qualitative TMJ landmarks definition

**Supplementary Material 2:** The intra-class correlation coefficient (ICC) results for intra- and inter-observer agreement of TMJ measurements

## Data Availability

The datasets used and analyzed during the current study are available from the corresponding author upon reasonable request.

## Abbreviations

TMD: Temporomandibular joint disorders
TMDG: Temporomandibular joint disorders group
TMD + MAG: Temporomandibular joint disorders and mandibular asymmetry group
BMD: Bone mineral density
S.S.: Stabilization splints
CBCT: Cone Beam Computed Tomography
3D: Three dimensions
MCD: Measures Condyle Displacement device
MIC: Maximum intercuspation
MRI: Magnetic Resonance Imaging
HU: Hounsfield Unit
AS: Anterior slope
SS: Superior slope
PS: Posterior slope
ADD: anterior disc displacement
DDwoR: disc displacement without reduction
DDwR: disc displacement with reduction
ID: internal derangement
