# Supplementary material for: Quantitative and qualitative condylar changes following stabilization splint therapy in patients with temporomandibular joint disorders with and without skeletal lateral mandibular asymmetry: a cone beam computed tomographic study
Source: BMC Oral Health. 2024 Mar 21;24:363. doi: 10.1186/s12903-024-04119-7 (PMC10956259; doi:10.1186/s12903-024-04119-7)
Supplement: Supplementary file 1 — Supplementary Material 1: Shows the quantitative and qualitative TMJ landmarks definition [file 12903_2024_4119_MOESM1_ESM.docx]

**Additional file 1**  Shows the quantitative and qualitative TMJ landmarks definition

| **Abbreviation** | **Definition** |
| --- | --- |
| **Quantitative landmarks** | |
| SF | The uppermost area of the glenoid fossa on the true horizontal line (THL) is used as a reference line. |
| SC | The superior-most mandibular condyle point in the sagittal section. The line perpendicular to the most superior aspects of the condyle (SC) was drawn from (SF). |
| AC | The anterior-most mandibular condyle point in the sagittal section. The line tangent to the most prominent anterior aspects of the condyle (AC) was drawn from (SF). |
| PC | The posterior-most mandibular condyle point in the sagittal section. The line tangent to the most prominent posterior aspects of the condyle (PC) was drawn from (SF). |
| CM | The medial point of the condyle in the coronal section. |
| CL | The lateral point of the condyle in the coronal section. |
| ACp | Most anterior condyle point are on an area of maximum convex curvature on the anterior side of the condyle in the sagittal section. |
| PCp | Most posterior condyle point are on an area of maximum convex curvature on the posterior side of the condyle in the sagittal section. |
| MCp | Most medial condyle point on an area of maximum convex curvature on the medial side of the condyle in the coronal section. |
| LCp | Most lateral condyle point are on the area of maximum convex curvature on the lateral side of the condyle in the coronal section. |
| P | The lowest point of the external auditory meatus. |
| C | The lowermost point of the articular tuberosity. |
| L | The line connects points C and P. |
| **Qualitative landmarks** | |
| AS | The ellipse region was selected on the anterior slope of the condyle in the sagittal section. |
| SS | The ellipse region was selected on the superior slope of the condyle in the sagittal section. |
| PS | The ellipse region was selected on the posterior slope of the condyle in the sagittal section. |
| MS | The ellipse region was selected on the medial point of the condyle in the coronal section. |
| LS | The ellipse region was selected on the lateral point of the condyle in the coronal section. |
