# Supplementary material for: Quantitative and qualitative condylar changes following stabilization splint therapy in patients with temporomandibular joint disorders with and without skeletal lateral mandibular asymmetry: a cone beam computed tomographic study
Source: BMC Oral Health. 2024 Mar 21;24:363. doi: 10.1186/s12903-024-04119-7 (PMC10956259; doi:10.1186/s12903-024-04119-7)
Supplement: Supplementary file 2 — Supplementary Material 2: The intra-class correlation coefficient (ICC) results for intra- and inter-observer agreement of TMJ measurements [file 12903_2024_4119_MOESM2_ESM.docx]

**Additional file 2** The intra-class correlation coefficient (ICC) results for intra- and inter-observer agreement of TMJ measurements

| TMJ measurements | Intraobserver | Interobserver |
| --- | --- | --- |
| Quantitative measurements | | |
| AJS (mm) | 0.944 | 0.958 |
| SJS (mm) | 0.916 | 0.931 |
| PJS (mm) | 0.939 | 0.874 |
| CMS (mm) | 0.944 | 0.907 |
| CLS (mm) | 0.935 | 0.937 |
| CL 1 (mm) | 0.947 | 0.915 |
| CL 2 (mm) | 0.938 | 0.907 |
| CH (mm) | 0.917 | 0.922 |
| HF (mm) | 0.935 | 0.906 |
| β (°) | 0.938 | 0.937 |
| θ (°) | 0.945 | 0.938 |
| β-θ (°) | 0.948 | 0.923 |
| Qualitative measurements | | |
| AS (Hu) | 0.932 | 0.922 |
| SS (Hu) | 0.922 | 0.886 |
| PS (Hu) | 0.952 | 0.924 |
| MS (Hu) | 0.903 | 0.919 |
| LS (Hu) | 0.912 | 0.943 |
